# Beyond Gestational Diabetes: Maternal and Offspring Health and Lifestyle 3 Years Postnatally in a Secondary Analysis of the UPBEAT Trial Cohort

**DOI:** 10.1111/ijpo.70076

**Published:** 2025-12-10

**Authors:** Claire Singleton, Danqi Zhuang, Kimberley Kavanagh, Kathyrn V. Dalrymple, Angela C. Flynn, Lucilla Poston, Claire L. Meek, Sara L. White

**Affiliations:** ^1^ University of Strathclyde Glasgow UK; ^2^ Department of Nutritional Sciences King's College London London UK; ^3^ School of Population Health Royal College of Surgeons in Ireland Dublin Ireland; ^4^ Department of Women and Children's Health School of Life Course and Population Sciences, King's College London London UK; ^5^ Leicester Diabetes Centre, University Hospitals Leicester Leicester UK; ^6^ Diabetes Research Centre University of Leicester Leicester UK; ^7^ Department of Diabetes Guys and St Thomas' NHS Foundation Trust London UK

**Keywords:** child health, gestational diabetes, lifestyle factors, obesity, postpartum, pregnancy

## Abstract

**Background:**

Gestational diabetes (GDM) is associated with increased future obesity risk in affected mothers and children.

**Objective:**

We assessed if dietary behaviours learnt during a GDM pregnancy positively impact maternal and child health 3 years postpartum.

**Method:**

In a secondary analysis, we included women with obesity recruited to the UPBEAT randomised controlled trial with 3‐year follow‐up postnatally (*n* = 441). Maternal and offspring anthropometry and dietary data were recorded antenatally and at follow‐up. Data were assessed using linear/logistic regression, adjusting for confounders.

**Results:**

Women with GDM (22%) had higher BMI (median 35.6 vs. 34.2 kg/m^2^; *p* = 0.049) and energy intake (1738.2 vs. 1551.6 kcal/day; *p* = 0.005) at ~16 weeks' gestation compared to unaffected women, but lower gestational weight gain (4.5 kg vs. 6.6 kg; *p* < 0.001). However, at 3 years postpartum BMI was similar between groups (35.8 vs. 35.2 kg/m^2^; *p* > 0.5). GDM‐exposed infants had a higher birthweight (55.4 vs. 45.9th centile; *p* = 0.008) than unexposed infants and at 3 years of age were more likely to be overweight/obese (International Obesity Task Force, IOTF, standards; OR 2.32; 95% CI 1.38, 3.91) but with similar skinfold thicknesses and dietary patterns.

**Conclusion:**

Women with GDM demonstrated reduced gestational weight gain, and despite a higher BMI than women without GDM in early pregnancy, this difference was not evident at 3 years postpartum. However, while maternal and offspring dietary behaviours were comparable between groups, exposed offspring were at increased risk of overweight/obesity at 3 years of age.

## 1 Introduction

Gestational diabetes (GDM), defined by new onset hyperglycaemia in pregnancy, affects approximately 14% of pregnancies worldwide with higher prevalence in women with obesity [1]. In the short term, hyperglycaemia increases the likelihood of increased fetal growth and adiposity leading to complications through labour and birth for both mother and baby [2]. Long‐term morbidity includes a ten‐fold greater risk of progression to Type 2 diabetes as well as a heightened risk of cardiovascular disease than those who are normoglycaemic through pregnancy [3, 4]. Offspring of women with GDM also have an increased risk of obesity and cardiometabolic disease [5] likely to result from persistent influences of hyperglycaemia on the developing fetus as well as dietary and physical activity behaviours during childhood and beyond [5].

Despite the known importance of the postnatal period, little is known about maternal and childhood lifestyle behaviours in women who develop GDM in the period after pregnancy. Breastfeeding is likely to reduce the risk of future Type 2 diabetes [6] and may be accompanied by weight loss in some women [7]. Advice delivered after a diagnosis of GDM consists of dietary change to improve food quality, with particular focus on carbohydrate type and quantity, and promotion of an increase in physical activity [1]. Whilst this educational advice may inform both short and long‐term behaviour change, little is known about the impact of diagnosis and education on postnatal health and lifestyle behaviours.

We have previously shown in a secondary analysis of the UK Pregnancies Better Eating and Activity Trial (UPBEAT [8]), a randomised controlled trial (RCT) of a behavioural intervention in women with obesity that following dietary advice, women with a diagnosis of GDM demonstrated greater reductions in energy and carbohydrate intake compared to women without GDM, and lower gestational weight gain post diagnosis [9]. We hypothesised that knowledge about dietary changes gained during pregnancy through the management of hyperglycaemia would also positively impact the postnatal health of mother and child, primarily through healthier lifestyle choices. We therefore aimed to assess the effect of a diagnosis of GDM in obese pregnancy on maternal and offspring dietary behaviours and child feeding choices, weight and anthropometric measures, at 3 years postpartum. This was undertaken in the UK using data from UPBEAT [8] and reported according to STROBE guidelines.

## 2 Methods

### 2.1 Study Design—Pregnancy Cohort

The UPBEAT trial was a multicentre RCT of a behavioural intervention addressing diet and physical activity versus standard antenatal care in pregnant women living with obesity between 2009 and 2014 (ISRCTN 89971375; REC 09/H0802/5), as described in detail elsewhere [8]. The intervention aimed to prevent GDM and large‐for‐gestational‐age (LGA) infants in 1555 pregnant women (> 16 years of age; BMI ≥ 30 kg/m^2^) with a singleton pregnancy recruited, through written informed consent, between 15 weeks and 18 weeks plus 6 days gestation. Women were excluded if they were unwilling or unable to give informed consent, or if they had a pre‐existing health condition. Women randomised to the intervention group received diet and physical activity advice alongside standard antenatal care. The control arm received standard antenatal care.

All participants undertook an oral glucose tolerance test (OGTT) (mean 27 weeks plus 6 days gestation) after at least 10 h of fasting. GDM was diagnosed based on the International Association of Diabetes and Pregnancy Study Groups (IADPSG) criteria as one or more of a fasting glucose ≥ 5.1 mmol/L, 1 h ≥ 10.0 mmol/L and 2 h ≥ 8.5 mmol/L, following a 75 g oral glucose load [10]. Individuals diagnosed with GDM were managed clinically according to local guidelines, receiving dietary and physical activity counselling (including reduction in foods high in carbohydrates and healthy choices) and pharmacological therapy (metformin and insulin) according to the degree of glycaemia. Pregnancy outcome data and neonatal measures were collected as described previously [8]. LGA was defined as > 90th centile on customised birthweight charts. The intervention did not significantly reduce the risk of GDM or LGA, nor impact the outcomes of this present analysis, thus allowing the data to be treated as a cohort for the current study.

Dietary intake was assessed by research midwives at study entry (between 15 and 18 weeks plus 6 days gestation), post intervention (between 27 and 28 weeks plus 6 days gestation) and in late pregnancy (between 34 and 36 weeks gestation). A 50‐item semi‐quantitative food frequency questionnaire (FFQ) adapted from the UK arm of the European Prospective Investigation into Cancer Study (EPIC) was used to assess dietary intake. The FFQ examined food and beverages consumed over the preceding month. An automated programme was developed to transform the data from the FFQ into daily nutrient intakes, details found elsewhere [11].

### 2.2 Study Design—Postnatal Cohort of Mothers and Offspring

**FIGURE 1:**
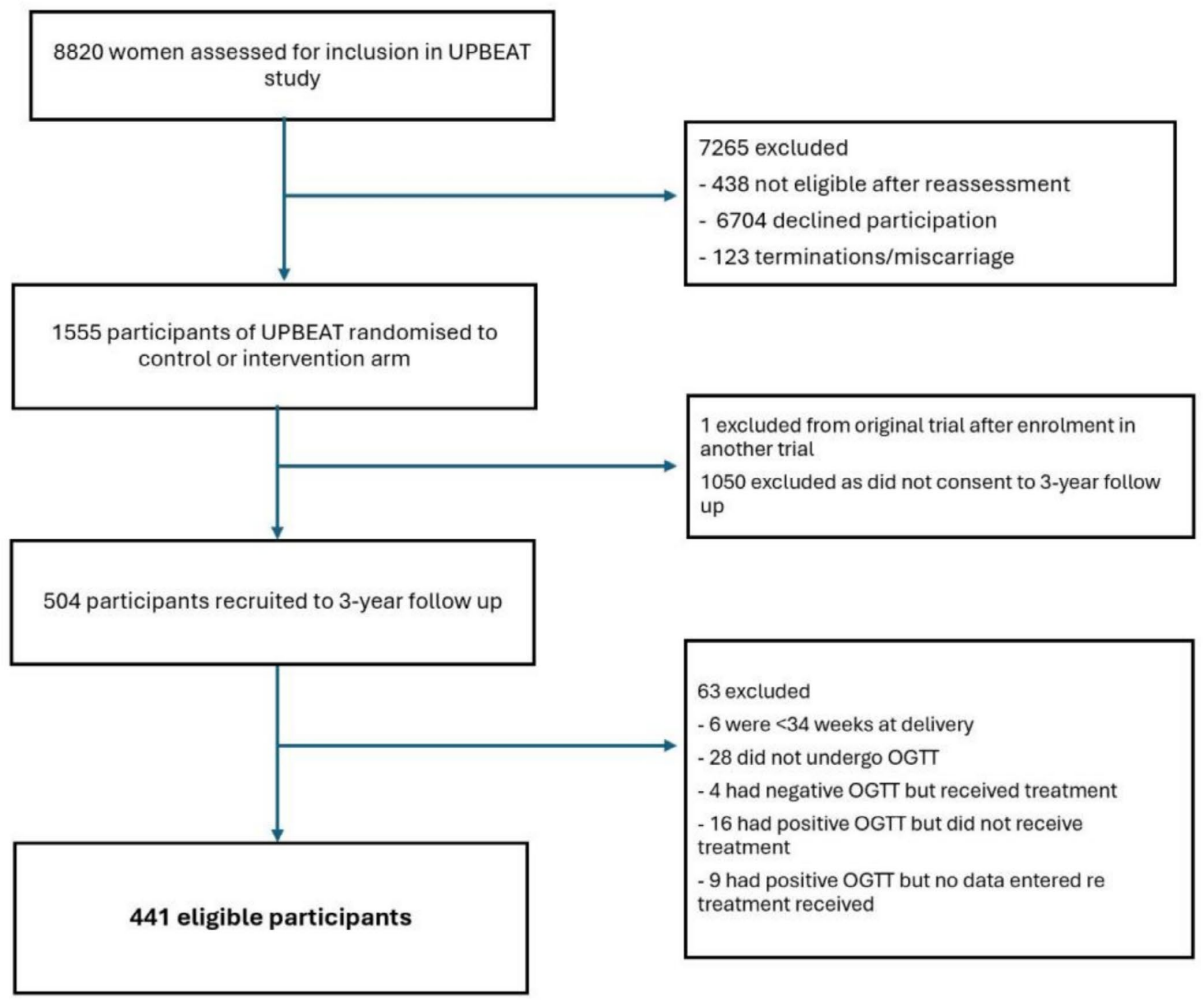
Consort diagram.

Participants in UPBEAT were invited to participate in a study visit 3 years post‐delivery; 504 mother‐child dyads attended the study visit and provided written informed consent (NHS Research Ethics Committee UK Integrated Research Application System; reference 12/LO/1108), as previously described [11]. Children were excluded from this analysis if they were born before 34 weeks' gestation or if they experienced severe illness (Figure 1).

### 2.3 Maternal Variables

Maternal height and weight were used to calculate BMI (kg/m^2^); height was assessed to the nearest 0.1 cm with a stadiometer (Harpenden; CMS Weighing Equipment Ltd.) and weight was measured to the nearest 0.1 kg with calibrated electronic scales (Seca), following removal of shoes and heavy clothing. Total gestational weight gain was derived from calculated pre‐pregnancy weight and last objective weight measured at ~36 weeks [8]. Maternal dietary intake after birth was assessed as during pregnancy, as described above. Mode of infant feeding at 4 months was recorded by maternal recall, and categorised as exclusively breastfeeding, exclusively formula feeding or mixed feeding.

### 2.4 Child Variables

Dietary intake in the child was assessed using a parent‐reported 85‐item food frequency questionnaire (FFQ) [11]. The FFQ assessed the frequency of consumption of the listed items in the previous 3 months. Factor analysis with orthogonal varimax rotation was used to derive dietary patterns of the children in order to provide a more comprehensive overview of dietary intake [11]. Three dietary patterns were identified and labelled “healthy/prudent” (characterised by brown bread, boiled and baked potatoes, rice and pasta, fish, vegetables, beans and pulses, fruit and nuts), “processed/snacking” (characterised by white bread, crisps and savoury snacks, roast potatoes, chips, processed foods, quiche and pizza, confectionary, desserts, cakes, biscuits, and low and high sugary drinks), and “African/Caribbean,” (characterised by yam/cassava/plantain, red meat, chicken and turkey, soups including African and Caribbean soups, rice/pasta, fish, and offal).

Child anthropometric outcomes assessed at the 3‐year study visit included; childhood overweight and obesity as defined by International Obesity Task Force (IOTF) sex‐specific BMI centiles; overweight—girls 89.3th, boys 90.5th, obese—girls 98.6th, boys 98.9th [11]; adiposity was measured by sum of skinfold thicknesses (sum of tricep, bicep, subscapular, suprailiac, and abdominal skinfolds measured in triplicate using Holtain skinfold callipers) and waist circumferences.

## 3 Statistical Analysis

Summary statistics and histograms of each variable, stratified by GDM were generated to visualise the distribution of the data. For comparison of the descriptive data, a two‐sample t‐test (or Wilcoxon rank sum test for skewed data) for continuous data, or Chi‐squared test for categorical data was employed as appropriate. A line plot of the median maternal BMI stratified by GDM diagnosis between baseline (15–18 weeks plus 6 days gestation) and 3 year follow up, plus intervening periods where available, was created to visualise the longitudinal relationship with GDM. Interaction tests were undertaken to assess any difference in the effect of the RCT intervention on study outcomes between GDM/no GDM groups. Variables were included as adjusters in multivariable analysis if there was published evidence to support an association with both GDM and the outcome. The multivariable analyses were undertaken as complete case analyses.

Multivariable linear regression models were constructed to estimate the association between GDM diagnosis and maternal BMI and dietary intake components, adjusting for potential confounding variables. Models were built for maternal outcomes at study entry (15–18 weeks plus 6 days gestation) and 3 years postpartum. Adjusters consisted of age, smoking status, index of multiple deprivation (IMD), ethnicity, parity, centre, intervention arm. The model of BMI at 3 years also included gestational weight gain as an adjuster, and women who were currently pregnant were excluded.

Multivariable logistic regression models were used to assess the differences in infant feeding modality and offspring adiposity between women with GDM and those without GDM. Multivariable linear regression modelling assessed the association between GDM diagnosis and offspring dietary pattern scores and offspring anthropometry at 3 years old. Maternal age, ethnicity, parity, IMD, smoking, maternal BMI, intervention arm, centre, and gestational weight gain were treated as adjusters in the models of infant feeding modality. The models of dietary patterns were adjusted for maternal age, ethnicity, parity, IMD, intervention arm, centre and infant sex. Anthropometry—birthweight customised centile adjusted for maternal age, IMD, intervention arm, centre and gestational weight gain. Child obesity at 3 years measured using International Obesity Task Force (IOTF) criteria and other anthropometric measures were adjusted for maternal age, maternal BMI at study entry, ethnicity, parity, IMD, intervention arm, centre. Anthropometric measures were also adjusted for infant sex. GDM treatment was not adjusted for in the models given that treatment lies on the causal pathway as a consequence of exposure to GDM.

## 4 Results

In this secondary analysis of the UPBEAT trial, 441 pregnant women with obesity who had an OGTT in pregnancy and attended the 3 year follow up visit were included, 22% of whom developed GDM. Interaction tests assessing difference in RCT intervention effect between GDM/no GDM participants were insignificant (all *p* > 0.05 see Tables S1 and S2). Maternal characteristics at study entry between groups (GDM vs. no GDM) were similar although women with GDM had higher maternal age and BMI, and lower gestational weight gain than women without GDM. Infants of women with GDM were born earlier, were lighter but had a higher birthweight customised centile. There were no significant differences in infant feeding modality at 4 months postpartum (Tables 1, 2, 3, 4).

**TABLE 1:** Baseline (15 weeks plus 0 days to 18 weeks plus 6 days' gestation) characteristics of eligible mothers, and children, stratified by GDM.

	No‐GDM *n* = 345 (78.2%)	GDM *n* = 96 (21.8%)	*p*
Maternal characteristics			
Maternal age (years)	31.1 (5.4)	32.8 (4.8)	0.006
Ethnicity			0.431
White	237 (69.0%)	61 (63.5%)	
Black	77 (22.3%)	24 (25.0%)	
Asian	13 (3.8%)	7 (7.3%)	
Other	18 (5.2%)	4 (4.2%)	
Index of multiple deprivation			0.676
1 (least deprived)	24 (7.0%)	4 (4.2%)	
2	28 (8.1%)	5 (5.2%)	
3	37 (10.7%)	12 (12.5%)	
4	124 (35.9%)	35 (36.5%)	
5 (most deprived)	129 (37.4%)	40 (41.7%)	
Missing values	3 (0.9%)	0 (0%)	
Current smoker	10 (2.9%)	6 (6.3%)	0.128
Nulliparous	165 (47.8%)	48 (50.0%)	0.728
Missing values	1 (0.3%)	0 (0%)	
Intervention group	173 (50.1%)	44 (45.8%)	0.502
GDM treatment			< 0.001
No GDM‐no treatment	345 (100%)	0 (0%)	
GDM‐diet only	—	29 (30.2%)	
GDM‐Insulin	—	14 (14.6%)	
GDM‐insulin and metformin	—	22 (22.9%)	
GDM‐metformin	—	31 (32.3%)	
Mode of delivery			0.854
LSCS in labour	68 (19.7%)	21 (21.8%)	
Operative vaginal	46 (13.3%)	11 (11.5%)	
Pre‐labour LSCS	63 (18.3%)	15 (15.6%)	
Unassisted vaginal	168 (48.7%)	49 (51.0%)	
Neonatal characteristics			
Male	174 (50.4%)	44 (45.8%)	0.490
GA at delivery (weeks)	40.1 (39.1, 41.1)	38.6 (38.1, 39.3)	< 0.001
Birthweight (g)	3507 (519.0)	3379 (414.8)	0.026
Customised birthweight centiles	45.9 (29.0)	55.4 (28.0)	0.004
LGA	29 (8.4%)	13 (13.5%)	0.167
SGA	43 (12.5%)	7 (7.3%)	0.213
Age at 3 years visit (months)	41.0 (39.0, 45.0)	42.0 (39.0, 45.0)	0.474

*Note:* Gestational weight gain recorded at 34–36 weeks' gestation, Neonatal characteristics determined at birth. *p* values from hypothesis test of differences: Two‐sample *t*‐test, Wilcoxon rank sum test and Chi‐squared test for symmetric, skewed and categorical data respectively.

Abbreviations: BMI, body mass index; GA, gestational age; GDM, gestational diabetes mellitus; LGA, large for gestational age (≥ 90th on customised birth centiles); LSCS, lower section caesarean section; SGA, small for gestational age (≤ 10th on customised birth centiles).

**TABLE 2:** Descriptives stratified by OGTT result (GDM and No GDM), and measures of effect size estimated from adjusted linear regression models between GDM status and measures of maternal BMI and diet over time (baseline (15 weeks plus 0 days to 18 weeks plus 6 days' gestation) and 3 years postpartum). Reference group is No GDM.

	No‐GDM *n* = 345 (78.2%)	GDM *n* = 96 (21.8%)	Adjusted *b* coefficient (95% CI)	*p*
	Median (IQR)	Median (IQR)		
Maternal anthropometry				
Baseline BMI (kg/m^2^)^a^	34.2 (32.5, 37.2)	35.6 (32.4, 38.4)	1.10 (0.00, 2.20)	0.049
BMI 3 years postpartum (kg/m^2^)^b^	35.2 (32.5, 39.9)	35.8 (32.6, 40.7)	0.46 (−1.11, 2.04)	0.567
Gestational weight gain^c^	6.6 (4.2)	4.5 (4.1)	−1.95 (−2.94, −0.96)	< 0.001
Maternal diet				
Energy (kcal) (baseline)^d^	1551.6 (1282.8, 1962.0)	1738.2 (1373.5, 2115.0)	275.16 (84.57, 465.75)	0.005
Energy (kcal) (3 year)^e^	1390.6 (1051.4, 1702.5)	1323.2 (1087.6, 1751.0)	29.34 (−107.99, 166.67)	0.675
Glycaemic load (baseline)^d^	114.1 (90.5, 150.1)	121.9 (92.9, 153.8)	12.29 (−2.34, 26.92)	0.099
Glycaemic load (3 year)^f^	108.5 (90.5, 137.2)	98.5 (81.8, 125.4)	−6.38 (−17.86, 5.09)	0.275
Glycaemic index (baseline)^d^	57.0 (4.2)^a^	56.3 (4.0)^a^	−0.63 (−1.67, 0.40)	0.230
Glycaemic index (3 year)^f^	57.3 (3.7)^a^	56.9 (3.9)^a^	−0.54 (−1.56, 0.48)	0.299

*Note:* *p* values from adjusted linear regression models. Adjusters for maternal anthropometry and diet: (i) = age, smoking status, IMD, ethnicity, parity, centre, intervention arm; BMI 3 years (i) plus GWG.

Abbreviations: BMI, body mass index; CI, confidence interval; GDM, gestational diabetes mellitus; GWG, gestational weight gain; IMD, index of multiple deprivation; IQR, interquartile range.

^a^ Adjusted regression sample size = 437.

^b^ Adjusted regression sample size = 379.

^c^ Adjusted regression sample size = 410.

^d^ Adjusted regression sample size = 378.

^e^ Adjusted regression sample size = 434.

^f^ Adjusted regression sample size = 321.

**TABLE 3:** Descriptives stratified by maternal OGTT result (GDM and No‐GDM), and measures of association estimated from adjusted logistic regression models between GDM status and infant feeding at 4 months and adiposity at 3 years (Reference group is No‐GDM).

	No‐GDM *n* = 345 (78.2%)	GDM *n* = 96 (21.8%)	Adjusted OR (95% CI)	*p* ^a^
	Total (percentage)	Total (percentage)		
Infant feeding at 4 months old^d^				
Exclusive breastfeeding	86 (24.9%)	21 (21.9%)		
Exclusive formula feeding	70 (20.3%)	24 (25.0%)	1.89 (0.85, 4.19)^b^	0.116
Mixed feeding	28 (8.1%)	13 (13.5%)	2.36 (0.91, 6.09)^b^	0.078
Missing values	161 (46.7%)	38 (39.6%)		
Offspring 3 years adiposity (IOTF)^e^				
Normal	229 (66.4%)	48 (50.0%)		
Overweight/obesity	101 (29.3%)	42 (43.8%)	2.32 (1.38, 3.91)^c^	0.0015
Missing values	15 (4.3%)	6 (6.3%)		

*Note:* Adjusters: (1) Infant feeding at 4 months old: Maternal age, ethnicity, parity, IMD, smoking, maternal BMI, intervention arm, centre, gestational weight gain. (2) IOTF: maternal age, maternal BMI, ethnicity, parity, IMD, intervention arm, centre.

Abbreviations: BMI, body mass index; CI, confidence interval; GDM, gestational diabetes mellitus; IMD, index of multiple deprivation; IOTF, international obesity task force.

^a^ *p* values from adjusted logistic regression models.

^b^ Exclusive breastfeeding as reference.

^c^ Normal as reference versus overweight/obesity.

^d^ Adjusted logistic model sample size = 236.

^e^ Adjusted logistic model sample size = 417.

**TABLE 4:** Descriptives stratified by maternal OGTT result (GDM and No‐GDM), and measures of effect size estimated from adjusted linear regression models between GDM status and measures of offspring anthropometry, and dietary pattern score at 3 years (Reference group is No‐GDM).

	No‐GDM *n* = 345 (78.2%)	GDM *n* = 96 (21.8%)	Adjusted *β* coefficient (95% CI)	*p* ^a^
	Mean (SD)/median (IQR)	Mean (SD)/median (IQR)		
Offspring 3 years dietary patterns *z*‐score				
Healthy/prudent^b^	−0.1 (−0.6, 1.2)	−0.1 (−0.5, 0.6)	0.17 (−0.04, 0.38)	0.108
Processed/snacking^c^	−0.1 (−0.7, 0.6)	−0.2 (−0.7, 0.6)	0.05 (−0.16, 0.27)	0.613
African/caribbean^d^	−0.2 (−0.6, 0.3)	−0.1 (−0.5, 0.6)	0.16 (−0.02, 0.33)	0.080
Offspring anthropometry				
Birthweight customised centile^e^	45.9 (29.0)	55.4 (28.0)	8.70 (2.26, 15.14)	0.008
3 years waist (cm)^f^	53.1 (3.9)	54.2 (4.8)	0.92 (−0.069, 1.91)	0.0682
3 years sum of skinfolds^g^ (mm)	40.2 (34.1, 49.2)	42.8 (33.9, 50.5)	4.00 (−0.14, 8.14)	0.0582

*Note:* Adjusters: (1)Dietary patterns: maternal age, ethnicity, parity, IMD, intervention arm, centre, infant sex. (2) Anthropometry: (2.1) birth weight customised centile: maternal age, IMD, intervention arm, centre, gestational weight gain. (2.2) 3 year anthropometric measures: maternal age, maternal BMI, ethnicity, parity, IMD, intervention arm, centre, infant sex.

Abbreviations: BMI, body mass index; CI, confidence interval; GDM, gestational diabetes mellitus; IMD, index of multiple deprivation; IQR, interquartile range.

^a^ *p* values from adjusted linear regression models.

^b^ Linear regression model sample size = 425.

^c^ Linear regression model sample size = 423.

^d^ Linear regression model sample size = 422.

^e^ Linear regression model sample size = 435.

^f^ Linear regression model sample size = 335.

^g^ Linear regression model sample size = 421.

### 4.1 Effect of GDM on Maternal Outcomes

At study entry, maternal BMI was 35.6 kg/m^2^ (IQR 6.1) in women with GDM which was higher than those without GDM (34.2 kg/m^2^; IQR 4.7, p 0.049), however there was no difference at 3 years postpartum. Total gestational weight gain was lower in women who developed GDM (6.6 kg; SD 4.2 vs. 4.5 kg; SD 4.1, *p* < 0.001). Trajectories of maternal BMI (median) are shown in Figure S1; a lower weight gain trajectory after diagnosis of GDM during pregnancy is evident; with suggestion that weight gain diverges 6 months after delivery, with higher weight gain in the GDM group. Women with GDM reported a significantly higher energy intake at study entry (1738.2 Kcal vs. 1551.6 Kcal) which was no longer evident at 3 years. No differences were observed in glycaemic load or glycaemic index at either timepoint between groups (Table 2).

### 4.2 Effect of GDM on Offspring Health at 3 Years of Age

The age of the children at the 3‐year follow‐up visit did not differ significantly between the GDM and non‐GDM groups (*p* = 0.474). Rates of overweight and obesity were higher according to IOTF categories at 3 years of age in the offspring of mothers who experienced GDM, although numbers were small overweight/obesity: 43.8% versus 29.3% (Overall OR 2.321.38, (1.38, 3.91), *p* = 0.0015; Table 3). There was no significant difference in waist circumference or sum of skinfold measures or in the difference in offspring dietary patterns between groups (Table 4).

## 5 Discussion

### 5.1 General Statement of Findings

We found evidence of a positive effect of GDM diagnosis and lifestyle education beyond pregnancy in this cohort of women with obesity. To our knowledge, this evidence of prolonged benefit has not been described previously. In contrast, children born to mothers with GDM had a significantly higher birthweight centile compared to those of women without GDM, and were more likely to be overweight and obese at 3 years of age.

### 5.2 Strengths and Limitations of the Study

The UPBEAT cohort is a well‐characterised study of multi‐ethnic women with obesity with robust processes and data analysis, uniquely positioned to assess the role of health behaviours on maternal and child health. Data were collected at multiple time points through pregnancy, and despite considerable loss to follow‐up [12], a cohort of 441 individuals and their children was included in this study from the post‐pregnancy phase through to 3 years postpartum. Compared to the original cohort, a higher percentage of individuals with GDM were included, and participants were older, had a lower BMI, were more commonly nulliparous, included fewer smokers, and were less deprived. There were however no differences between offspring descriptors at birth and those included at the 3‐year follow‐up. We note that fewer individuals were included in some postpartum analyses due to this being a complete case analysis. Individuals who had interim pregnancies were not excluded from the analyses at 3 years; only those who were concurrently pregnant, and through postpartum weight retention this might impact upon maternal BMI.

Strengths include data collection at specific timepoints enabling inferences about trajectories from pregnancy to 3 years postpartum. Anthropometric measurements were carried out in triplicate after training. A limitation is that dietary data were collected through self‐report, a method commonly associated with underreporting. Also no data were available regarding the development or management of hyperglycaemia postpartum.

Longitudinal or SEM‐based mediation analyses of maternal data were precluded due to a lack of power when undertaking complete case analyses across time. As offspring dietary patterns were only assessed at age 3 years, this precluded the possibility of formally modelling trajectories. As a result cross‐sectional associations only were explored.

### 5.3 Meaning of Study Results

#### 5.3.1 Maternal Weight and Lifestyle Behaviours

Data from the UPBEAT cohort documented here and previously [9] show the diagnosis of GDM and subsequent dietary behaviour change was associated with reduced gestational weight gain compared with women with no GDM. This difference, if through maternal fat accrual, may have had an enduring impact at 3 years postnatally, as the BMI at this follow‐up visit was comparable to women without GDM. This contrasted with early pregnancy when BMI was higher in the GDM group. Reduced pregnancy weight gain therefore appeared to have had a sustained effect by narrowing the weight difference between groups. This is consistent with Mamun and colleagues [13] who demonstrated that gestational weight gain continues to impact women's weight even 2 decades after the index pregnancy.

The higher energy intake at study entry between GDM and non‐GDM groups, no longer significant at 3 years postpartum, may also denote a sustained reduction in energy intake in the GDM group, contributing to weight equivalence between groups at 3 years. The lack of impact on breastfeeding (although not universally discussed as part of GDM care), and on further postpartum weight differences and other dietary measures highlights the challenges of long‐term change in women with GDM with a recent birth, and caring responsibilities. This has also been evidenced by the poor uptake [14] of diabetes prevention programmes after pregnancy. Proposed barriers to sustained behaviour change in GDM include a feeling of abandonment by healthcare professionals after pregnancy [15], and lack of income and social support [16]. Our data suggest the need for novel targeted interventions after 6 months postnatally as women who had experienced GDM appeared to gain weight beyond this point (Figure S1).

#### 5.3.2 Offspring BMI and Adiposity

Offspring exposed to GDM were more likely to have overweight or obesity at 3 years of age, despite comparable diet and comparable postnatal exposure to maternal obesity. This confirms previous findings that intrauterine exposure to maternal hyperglycaemia has an ongoing effect especially when coupled with maternal obesity [17, 18]. Our data add to this literature through analysis of offspring diet, which showed no difference between groups, although we recognise limitations of the indirect methods used, for example parental recall of diet over the previous 3 months.

Although there was an increase in overweight/obesity, we demonstrated no statistical differences in anthropometry and skinfold data in the children between GDM and non‐GDM groups. This may reflect a lack of power, or the inherent variability in anthropometric measurement [19] as well as limitations of the methods used. Future work in larger cohorts using more accurate methods of fat mass such as whole‐body plethysmography [20, 21] would provide greater insight.

### 5.4 Policy Implications

With rising rates of obesity and GDM in the maternal population, there is an urgent need to improve outcomes in offspring in order to prevent the trans‐generational cycle of obesity and metabolic disease [22]. Communication of long‐term risks in pregnancy is central to promoting a lifecourse approach to women's health, and that of their children. Large‐scale, longer‐term strategies, that begin before the preconception period, are needed to improve maternal and child health across the lifecourse [23, 24]. Such approaches do not rely upon fortitude in the postnatal period, a time when there are unique challenges to following a healthy lifestyle.

## 6 Conclusions

Women with GDM demonstrated reduced gestational weight gain, and despite a higher BMI and energy intake than women without GDM in early pregnancy, this difference was not evident at 3 years postpartum. However, offspring born to mothers with GDM were at increased risk of overweight/obesity at 3 years of age.

## Author Contributions

S.L.W. and C.L.M. were responsible for the conceptualisation, design and methodology, data interpretation and wrote and revised the written report. C.S. and D.Z. carried out the statistical analysis under the supervision of K.K., and together wrote the results section, created the tables and figures, and read and revised the final manuscript. K.V.D. and A.C.F. contributed to data collection, study design and analysis, particularly of dietary and lifestyle data from postnatally to 3 years in mothers and children and read and revised the final manuscript. L.P. had overall responsibility for the conceptualisation, design, methodology, funding acquisition, data analysis and reporting of the original UPBEAT study, contributed to the design and conceptualisation of the current analysis and read and revised the final manuscript. All authors reviewed the final version before submission.

## Funding

This work was supported by the National Institute for Health and Care Research (RP‐PG‐0407‐10452), Seventh Framework Programme (FP7/2007‐2013, 289346), Medical Research Council (MR/L002477/1), Chief Scientist Office, Scottish Government Health Directorates (Edinburgh) (CZB/A/680), Biomedical Research Centre at Guys & St Thomas NHS Foundation Trust & King's College London, British Heart Foundation (FS/17/71/32953), Tommy's Charity (SC039280).

## Conflicts of Interest

The authors declare no conflicts of interest.

## Supporting information


**Data S1:** ijpo70076‐sup‐0001‐Supinfo.pdf.

## Data Availability

The data that support the findings of this study are available on request from the corresponding author. The data are not publicly available due to privacy or ethical restrictions.
