# Supplementary material for: Beyond Gestational Diabetes: Maternal and Offspring Health and Lifestyle 3 Years Postnatally in a Secondary Analysis of the UPBEAT Trial Cohort
Source: Pediatr Obes. 2025 Dec 10;21(1):e70076. doi: 10.1111/ijpo.70076 (PMC12690341; doi:10.1111/ijpo.70076)

Supporting Information for:

Beyond Gestational Diabetes: Maternal and Offspring Health and Lifestyle 3 years Postnatally in a Secondary Analysis of the UPBEAT Trial Cohort

Claire Singleton[1*], Danqi Zhuang[1*], Kimberley Kavanagh[1], Kathyrn V Dalrymple[2], Angela C Flynn[3], Lucilla Poston[4], Claire L Meek[5,6^], Sara L White[4,7^]

Affiliations:
[1]University of Strathclyde, Glasgow, UK
[2]Department of Nutritional Sciences, King's College London, London, UK
[3]School of Population Health, Royal College of Surgeons in Ireland, Dublin, Ireland
[4]Department of Women and Children's Health, School of Life Course and Population Sciences, King's College London, London, UK
[5]Leicester Diabetes Centre, University Hospitals Leicester, Leicester, UK
[6]Diabetes Research Centre, University of Leicester, Leicester, UK
[7]Department of Diabetes, Guys and St Thomas' NHS Foundation Trust, London, UK
*Joint first authors, ^joint last authors.

Correspondence and reprint requests can be made to Dr Sara L White, Department of Women and Children's Health, School of Life Course and Population Science, King's College London, London SE7 1EH, UK.
Email: sara.white@kcl.ac.uk

Supplementary Table 1- Interaction test between GDM and intervention arm (maternal variables)

**MATERNAL ANTHROPOMETRY**	***p- value***[a]
**Baseline BMI (kg/m^2) (n=441)**	0.1343
**BMI 3 years postpartum (kg/m^2) (n=408)**	0.213
**Gestational Weight Gain (n=414)**	0.4979
**MATERNAL DIET**	
**Energy (kcal) (Baseline) (n=381)**	0.0647
**Energy (kcal) (3 year) (n=438)**	0.5169
**Glycaemic Load (Baseline) (n=381)**	0.1618
**Glycaemic Load (3 year) (n=325)**	0.3205
**Glycaemic Index (Baseline) (n=381)**	0.491
**Glycaemic Index (3 year) (n=325)**	0.3572

Linear regression models of maternal anthropometry or diet as outcome against fixed effects of GDM diagnosis, intervention arm, and the interaction of GDM diagnosis and intervention arm. a. The reported p-values are those of the interaction.

Supplementary Table 2 - Interaction test between GDM and intervention arm (offspring variables)

**OFFSPRING VARIABLES**	***P- value***[a]
**Infant feeding at 6 months old (n-242)**	
Exclusive breastfeeding (as reference)	
Exclusive formula feeding	0.880
Mixed feeding	0.822
**Offspring 3y adiposity (IOTF) (n=420)**	
Underweight (1)	0.073[b]
Normal (2)	
Overweight (3)	
Obesity (4)	
Morbid obesity (5)	
**Offspring 3y dietary patterns z-score**	
Healthy/Prudent (n=427)	0.459
Processed/Snacking (n=426)	0.901
African/Caribbean (n=425)	0.286
**Offspring anthropometry**	
Birthweight customised centile (n=441)	0.314
3y BMI for age z-score (n=431)	0.068
3y Waist (cm) (n=424)	0.211
3y Sum of skinfolds (mm) (n=336)	0.174

Linear regression / logistic models of offspring anthropometry or diet or feeding modes as outcome against fixed effects of GDM diagnosis, intervention arm, and the interaction of GDM diagnosis and intervention arm.

a. The reported p-values are those of the interaction.
b. 1+2 as reference vs 3+4+5

Supplementary Figure 1: Line plot of longitudinal changes in median maternal BMI (kg/m^2) stratified by GDM diagnosis from the baseline visit (~16 weeks) through to 3-years postpartum. Blue line: GDM, Red line: No GDM

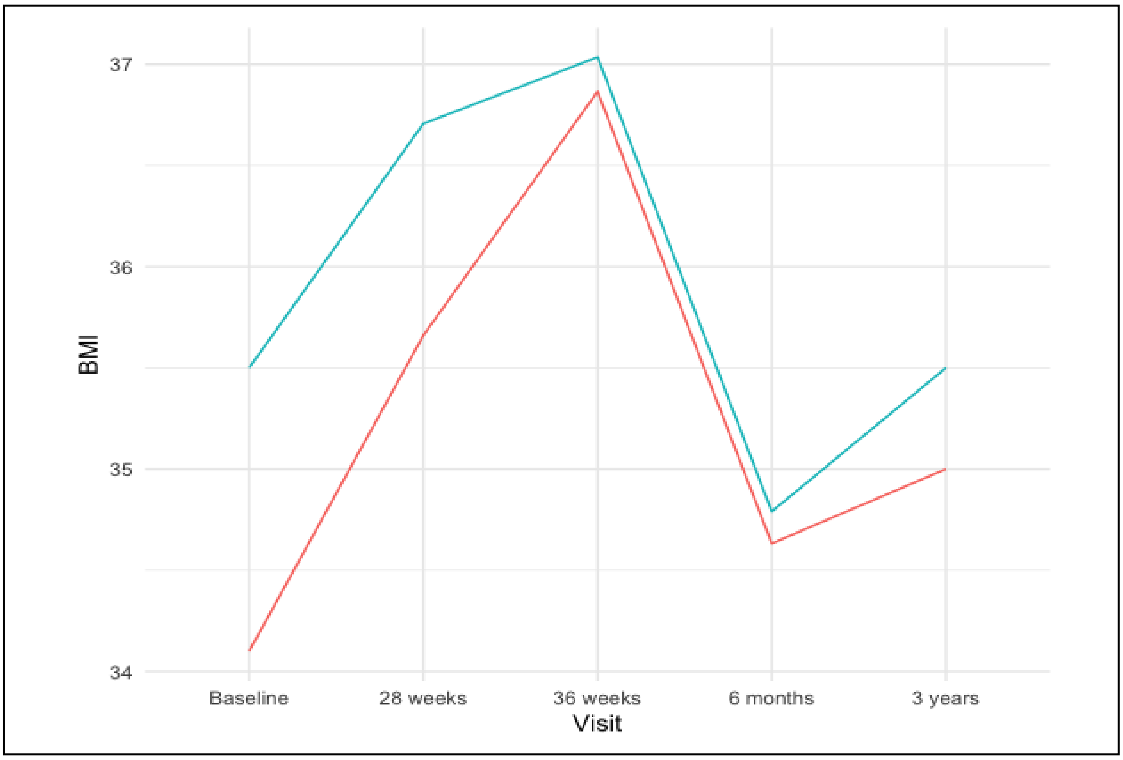

Supplement: Supplementary file 1 — Data S1: ijpo70076‐sup‐0001‐Supinfo.pdf. [file IJPO-21-e70076-s001.pdf]
